# An Artificial Intelligent Signal Amplification System for *in vivo* Detection of miRNA

**DOI:** 10.3389/fbioe.2019.00330

**Published:** 2019-11-21

**Authors:** Xibo Ma, Lei Chen, Yingcheng Yang, Weiqi Zhang, Peixia Wang, Kun Zhang, Bo Zheng, Lin Zhu, Zheng Sun, Shuai Zhang, Yingkun Guo, Minmin Liang, Hongyang Wang, Jie Tian

**Affiliations:** ^1^CAS Key Laboratory of Molecular Imaging, Institute of Automation, Chinese Academy of Sciences, Beijing, China; ^2^School of Artificial Intelligence, University of Chinese Academy of Sciences, Beijing, China; ^3^International Co-operation Laboratory on Signal Transduction, Eastern Hepatobiliary Surgery Institute, Second Military Medical University, Shanghai, China; ^4^National Laboratory of Biomacromolecules, CAS Center for Excellence in Biomacromolecules, Institute of Biophysics, Chinese Academy of Sciences, Beijing, China; ^5^Department of Radiology, West China Second University Hospital, Sichuan University, Chengdu, China; ^6^Experimental Center of Advanced Materials School of Materials Science & Engineering, School of Materials Science & Engineering, Beijing Institute of Technology, Beijing, China; ^7^Beijing Advanced Innovation Center for Big Data-Based Precision Medicine, Beihang University, Beijing, China

**Keywords:** *in vivo* detection of non-coding RNA, an artificial intelligent signal amplification system, early diagnosis of precancerous lesions, fluorescent molecular tomography, stem cell tracing

## Abstract

MicroRNAs (miRNA) have been identified as oncogenic drivers and tumor suppressors in every major cancer type. In this work, we design an artificial intelligent signal amplification (AISA) system including double-stranded SQ (S, signal strand; Q, quencher strand) and FP (F, fuel strand; P, protect strand) according to thermodynamics principle for sensitive detection of miRNA *in vitro* and *in vivo*. In this AISA system for miRNA detection, strand S carries a quenched imaging marker inside the SQ. Target miRNA is constantly replaced by a reaction intermediate and circulatively participates in the reaction, similar to enzyme. Therefore, abundant fluorescent substances from S and SP are dissociated from excessive SQ for *in vitro and in vivo* visualization. The versatility and feasibility for disease diagnosis using this system were demonstrated by constructing two types of AISA system to detect Hsa-miR-484 and Hsa-miR-100, respectively. The minimum target concentration detected by the system *in vitro* (10 min after mixing) was 1/10th that of the control group. The precancerous lesions of liver cancer were diagnosed, and the detection accuracy were larger than 94% both in terms of location and concentration. The ability to establish this design framework for AISA system with high specificity provides a new way to monitor tumor progression and to assess therapeutic responses.

## Introduction

Foundational to physiological programs in developmental and disease contexts is microRNA (miRNA) regulation, especially for cancer; miRNAs have been confirmed to be oncogene drivers or inhibitory factors (Anastasiardou et al., 2018). When cells become cancerous, they secrete special proteins that can be used for targeted imaging. However, the expression levels of miRNAs in cells are often changed before they become cancerous. Compared to detecting proteins secreted by tumors, detecting secreted miRNAs has become more attractive for monitoring tumor progression. The molecular biology principle of complementary base paring for miRNA facilitates the design of a universal, highly sensitive detection system that can visualize miRNA *in vivo* to monitor tumor progression. Based on this principle and the fluorescence quenching principle, molecular beacons have been synthesized with DNA or RNA base sequences (Yurke et al., 2000; Seelig et al., 2006; You et al., 2015, 2017; Ma et al., 2017; Fu et al., 2018), enabling their application in the detection of target RNA *in vitro* and at the cellular level (Yurke et al., 2000; Sawada et al., 2009; Auslander and Fussenegger, 2014; Green et al., 2014; Koc et al., 2015; Parolini et al., 2016; Yang et al., 2016a; Zhang et al., 2017; Li et al., 2018).

However, these reported strategies cannot achieve *in vivo* visualization of miRNA (not to mention miRNA quantification) because the fluorescence generated by the molecular beacon is not amplified; simple conjugation with one unit of target miRNA only release one unit of the fluorescent molecule. In a short period of time, chemical reactions often cannot be completely carried out, so a unit of target molecules often cannot produce a unit of fluorescent molecules. Amplification of fluorescent signal would produce a sufficient signal to meet the needs of *in vivo* detection. Recently, some amplification strategies were successfully used in logic circuits and for *in vitro* detection of the Zika virus (Pardee et al., 2016); however, these strategies cannot be directly applied to *in vivo* monitoring of miRNA because they do not adequately account for the chemical reaction that occurs within the probe itself and its *in vivo* degradation (Green et al., 2014; Hall and Macdonald, 2016; Pardee et al., 2016).

Despite its fundamental importance and theoretical achievability, as well as the aforementioned promising early efforts (Yurke et al., 2000; Seelig et al., 2006; Siuti et al., 2013; Li et al., 2016; Ma et al., 2017; You et al., 2017), it remains challenging to develop a general strategy for designing and synthesizing an artificial intelligent signal amplification (AISA) system [including double-stranded SQ (S, signal strand; Q, quencher strand) and double-stranded FP (F, fuel strand; P, protect strand)] that can react with the miRNA of interest and further amplify the generated signal (due to double-stranded FP) for the purpose of *in vivo* detection. The key design challenge is to barely achieve a reaction between the two components in the AISA system to obtain as little noise as possible in the absence of the target miRNA and to initiate a cascade in the presence of the target miRNA to obtain as many useful signals as possible. Fortunately, Song et al. designed a probe for the detection of miR-21 (Wang et al., 2017), which was the structural prototype of the amplification system and initially realized the increase in signal. However, the specificity is not considered in the design of the system and the nonspecific signal is strong, so the fluorescence of miR-21 was only detected at the tissue level. The AISA system contains two double-stranded DNA, SQ and FP, and there are many combinations of SQ and FP (kinds of terminal α, β, and γ) that can meet the corresponding three-step amplification response. Our main innovation lies in that, through experiments, we have selected the AISA system with the optimal signal/noise ratio (SNR). Meanwhile, we will further explore the relevant conditions of the AISA system with the optimal SNR in the future research.

In addition, an appropriate carrier is indispensable to transport the active components and guarantee that the detection reaction occurs. A suitable carrier must satisfy the following three basic requirements. First, during the transportation process after injection via the tail vein, the carrier of the AISA system should load as many active components as possible and resist degradation by various enzymes in the body to achieve a sufficient concentration in the target organs. Second, after reaching the target organ, the AISA system should be able to release its active components, that is, two double-stranded DNA molecules, which should cross the cell membrane and enter the cytoplasm. Third, and most critically, the cytoplasmic concentration of the active components released and transported by the AISA system must be sufficient to guarantee an effective detection reaction, and these components should be able to distinguish the target miRNA and its analogs effectively so that we can obtain an image with a high signal/noise (SNR) ratio. In the previous studies, many researchers have innovatively prepared vectors to deliver small interfering RNA, miRNA, and immunotherapeutic antibody for molecular therapy (Zhong et al., 2015; Wang et al., 2018; Yaghini et al., 2018; Liu et al., 2019; Wu et al., 2019; Xu et al., 2019). These carriers can transport the substances into cells, but their transport efficiency varies, which will affect subsequent calculation of the relationship between fluorescence intensity and RNA concentration. Therefore, the mature carrier with stable delivery efficiency is the best choice for our study.

In this study, we designed the AISA system including two double-stranded DNAs (SQ and FP) for *in vivo* visualization of miRNA and clarified the reaction principle. We verified the detection sensitivity and specificity of the system *in vitro*. The versatility and practicability of this system were demonstrated by constructing two types of AISA system to detect Hsa-miR-484 and Hsa-miR-100, respectively. Based on this detection system, the precancerous lesions of liver cancer were diagnosed and reconstructed.

## Materials and Methods

### Materials

All chemical reagents were purchased from Sigma-Aldrich (Sigma-Aldrich, Beijing, China). Lipofectamine 3000 reagent (included lipo3000 and reagent) were purchased from ThermoFisher (USA). LO2 cells were purchased from the National Infrastructure of Cell Line Resource, and miR-484-transfected LO2 normal human liver cells (LO2-miR-484) were provided by Dr. Lei Chen from the Second Military Medical University. All animals were purchased from Beijing Vital River Laboratory Animal Technology Co., Ltd., and all animal experiments were conducted in accordance with the guidelines of the Institutional Animal Care and Use Committee at Peking University and the Eastern Hepatobiliary Surgery Hospital Research Ethics Committee.

### Standard Buffer Conditions

All double- and single-stranded DNA molecules were suspended and stored in Tris–HCl buffer (150 mM NaCl pH balanced to 7.4, with 1.25 mM MgCl_2_) at 4°C. This buffer concentration produces 303.75 osmotic pressure, which is near that of the internal environment.

### DNA Concentration

Nucleotide concentrations were calculated according to the molar extinction coefficient of single-stranded DNA. The measured extinction value at 260 nm and ultraviolet–visible spectra were obtained through a Cary UV-300 ultraviolet–visible spectrophotometer.

### Preparation of Double-Stranded SQ and FP

Each strand was prepared with nominal stoichiometry at 100 μM concentration in Tris–HCl buffer. The signal strands S were mixed with quencher strands at a 6:7 molar ratio to ensure that the signal strands were saturated by quencher strands and suppressed the background noise without miRNA. Single-stranded F was mixed with single-stranded P at a 1:1 molar ratio. The two mixtures were heated at 90°C for 10 min and then slowly cooled to room temperature (more than 2 h). The double-stranded SQ and FP were stored at 4°C for further use.

### Preparation of the AISA System

Then, 100 μl of double-stranded SQ (50 μM), 200 μl of double-stranded FP (50 μM), and 5 μl of reagent (included in the Lipofectamine 3000 kit) were mixed homogenously. After 5 min, 5 μl of Lipo3000 was added to the mixture to construct the AISA system.

### *In vitro* Time-Based Fluorescence Studies

Fluorescence emission signals were acquired using an F-7000 fluorescence spectrophotometer (Hitachi High-Tech Science Corporation, Tokyo, Japan) with 649 nm excitation and 669 nm emission wavelengths for Cy5. The fluorescence intensity of 100 nM single-stranded S was set as 1,000, and the fluorescence intensity of others was normalized to that of S.

### High-Speed Atomic Force Microscopy Imaging

First, 1 μl of sample containing single-stranded miR-484 (100 μM), double-stranded SQ, and double-stranded FP (50 μM) was diluted with 1 μl of 100 mM NiCl_2_ and 8 μl of Milli-Q water (final SQ/FP: 5 μM, 10 mM NiCl_2_). Two microliters of diluted DNA was dropped on cleaved mica. After 10 min, a high-speed atomic force microscopy instrument (Research Institute of Biomolecule Metrology Co., Ltd, Japan) (Kodera et al., 2010; Katan and Dekker, 2011; Miyagi et al., 2016; Shibata et al., 2017) was used to image DNA in Milli-Q water. The conditions were set as follows: cantilever, BL-AC10DS-A2 (Olympus, Japan); resolution, 200 × 200 pixels; mode, ac mode in liquid. All images were analyzed using ImageJ (NIH) SPIP (Image Metrology A/S).

### Detection of miR-484 and miR-100 at the Subcellular Level

The LO2-miR-484/MSC-miR-100 cells were seeded at ≈1 × 10^4^ cells per well into uncoated and glass-bottomed confocal plates, and the seeded cells were incubated with the AISA system (100 nM) for 4 h in Dulbecco's modified Eagle medium (HyClone, Thermo Fisher Scientific, USA) and growth medium [10% FBS (Gibco), 37°C, 5% CO_2_]. Fluorescein isothiocyanate-phalloidin was diluted 200 times with phosphate-buffered saline (PBS), added to the cells, and incubated for 40 min. For dynamic fluorescence confocal observation, 4′,6-diamidino-2-phenylindole was diluted 400 times with PBS and added to the cells for the subsequent image acquisition.

### *In vivo* Detection of Injected miR-484 Based on miR-484-Injected Animal Models

Three types of animal models were prepared for evaluation of the AISA^484^ system. First of all, 25 μl of miR-484-Lipo solution (5 μl of reagent and 5 μl of Lipo3000 per 300 μl solution) at 20 μM was injected into C57 mouse livers with a 2.5-mm injection depth. Then, the mice were injected with AISA system solution (100 μl per 20 g) via the tail vein, and fluorescence images were acquired at 22 min using the fluorescence system that we developed (**Figure 3**).

To evaluate the ability of the AISA system to distinguish miR-484 from tumors, miR-484-injected and diethyl nitrosamine (DEN)-induced tumor-bearing mouse models were generated. First, 4 mg/ml DEN was injected into the abdomen of a 15-day-old mouse at a dose of 25 mg/kg. Seven months later, after opening the abdominal cavity, 25 μl of miR-484 solution was injected into mouse liver at an injection depth of 4 mm, where no tumor formation had occurred. Fluorescence images were also acquired using our system under the following conditions: binning = 2, exposure time = 0.2 s, excitation wavelength = 649, emission wavelength = 680 (Figure S4). A multiple target model was established by injecting miR-484 into the rat liver at 3- and 2-mm intervals (Figure S5).

To evaluate the universality of this design strategy for the AISA system, we redesigned the AISA system to detect miR-100 and generated a miR-100 mouse model to evaluate the detection ability. A total of 25 μl of miR-100 solution of 60, 40, and 10 μM was injected into the femoral head of the BALB/c mice with a lateral approach. The mice were injected with 100 μl of AISA^100^ system solution via the tail vein, and fluorescence images were then acquired at 5, 12, 22, 32, and 60 min using our system (Figure S9). Since miR-100 is an important indicator of the progression of gastric cancer, we also established a high-expression miR-100 model of gastric cancer to evaluate the detection capability of the AISA system. SGC-7901-miR-100 tumor-bearing mice were generated by subcutaneous injection of 10^6^ cells into the right upper flanks of BALB/c nude mice. The mice were imaged to detect miR-100 when the tumor volume was <10 mm^3^, after ~10 days. The mice were treated with 100 μl of AISA^100^ system solution via the tail vein, and fluorescence images were acquired at 15, 40, and 60 min and 2, 6, and 12 h using the fluorescence imaging system under the same conditions as above (**Figure 3D**).

### *In vivo* Detection of miR-484 With DEN-Induced Mice and Human Samples

In addition, to evaluate the practicability of the AISA system, 4 mg/ml DEN was injected into the abdomen of the 15-day-old mice at a dose of 25 mg/kg to generate a tumorigenic model with high expression of miR-484. After ~3 months, the mice were treated with 100 μM AISA^484^ (included SQ and FP) system at a dose of 100 μl/kg via the tail vein, and fluorescence images were acquired using our system (**Figure 4A**). Three-dimensional (3D) information of miRNA were reconstructed using fluorescence molecular tomography based on sparsity adaptive correntropy matching pursuit method (Zhang et al., 2018b). Then, to evaluate the expression of miR-484, reverse transcription PCR (RT-PCR) was performed on the samples dissected from the mouse liver according to the location of fluorescence spots on the surface and the depth information derived from the 3D reconstructed results.

For detection of miR-484 in human samples, the samples were washed three times with PBS. Then, the samples were immersed in the 100 μM AISA^484^ solution for ~15 min. Then, the samples were removed, and the floating liquid was wiped off and placed in the fluorescent imaging instrument developed by ourselves. The imaging parameters were set as: exposure time = 1 s, binning = 4, excitation wavelength = 649, and emission wavelength = 680.

### *In vivo* Reconstruction of Injected and Transfected miR-484

The surface fluorescence signal detected by our system is related to the following two processes: the reaction between miRNA and double-stranded SQ and FP in the cell leads to fluorescence; the fluorescence signal passes through a certain depth of tissue to the body surface and is acquired by our system.

When a fluorescence signal is generated inside the body, we cannot directly detect the fluorescence intensity; we can only detect the distribution and intensity of fluorescence that passes through the tissue and reaches the body surface. The transmission of fluorescence *in vivo* obeys a certain rule, which can be expressed by the following equation describing the propagation of light (Ma et al., 2013; Xue et al., 2013; Qin et al., 2014; Ye et al., 2014; Zhang et al., 2018b):

$$\begin{array}{l} \nabla \left[D_{x}\left(r\right)\nabla \Phi_{x}\left(r\right)\right]-\mu_{ax}\left(r\right)\Phi_{x}\left(r\right)=-\Theta \delta \left(r-r_{l}\right)\left(r\in \Omega \right) \end{array}$$

$$\begin{array}{l} \nabla \left[D_{x}\left(r\right)\nabla \Phi_{x}\left(r\right)\right]-\mu_{am}\left(r\right)\Phi_{m}\left(r\right)={-\Phi }_{x}\left(r\right)\eta \mu_{af}\left(r\right)\left(r\in \Omega \right) \end{array}$$

$$2{\text{D}}_{\text{x},\text{m}}\left(\text{r}\right)\nabla \Phi_{\text{x},\text{m}}\left(\text{r}\right)+\text{q}\Phi_{\text{x},\text{m}}\left(\text{r}\right)=0(\text{r}\in \partial \Omega )$$

where *r* denotes the nodes inside the problem domain Ω; *r*_*l*_ is the position of point excitation sources, which are placed on one mean free path of photon transport beneath the surface of Ω; μ_*ax,am*_ and μ_*sx,sm*_ denote the absorption and scattering coefficients, respectively; Θ is the excitation intensity; and Φ(*r*) is the photon flux density at node *r*. ημ_*af*_(*r*) is the fluorophore distribution to be reconstructed, where η is the quantum yield and *q* denotes the optical reflective index. In the process of fluorescence tomography reconstruction, we used Robin boundary conditions to calculate the position of fluorescence source *in vivo* (Ma et al., 2013). For nearly 10 years, our research group has been working on 3D reconstruction of internal fluorescence and developed series of algorithms. Thus, according to Equation (1), the 3D distribution of the inner source can be reconstructed.

### Toxicity of the AISA System

To evaluate the toxicity of the AISA system, 2 × 10^3^ LO2 normal human liver cells per well were seeded into 96-well plates overnight. The cells were treated with seven concentrations of the AISA system (0, 1, 5, 10, 20, 40, 60, and 100 nM) for 24 h. Then, 10 μl of CCK-8 was added to each well. After incubation for 2 h at 37°C, the absorbance was measured at 450 nm using a BioTeK Synergy HT Universal Microplate Reader.

To evaluate the toxicity of the AISA system *in vivo*, 21 mice were randomly divided into three groups. Two of the groups were treated with 100 μl of the AISA system via the tail vein, and one group was treated with the same amount of PBS. For the mice in the two groups administered the AISA system, blood and tissue, including liver, muscle, heart, spleen, kidney, stomach, lung, brain, and skin tissue, were collected 1 and 2 weeks later, respectively. For the mice in the control group, serum and tissues were taken 2 weeks later. With the serum samples, creatine kinase (CK) and lactate dehydrogenase (LDH) were measured to evaluate cardiac function (Prakash, 1978; Yong et al., 2002); aspartate transaminase (AST), alanine aminotransferase (ALT), and alkaline phosphatase (ALP) were measured to evaluate liver function (Kory et al., 1959); and blood urea nitrogen (BUN) and creatinine (CREA) were measured to evaluate kidney function (Griebling, 2017). The tissue samples were embedded in wax blocks, sectioned and stained with hematoxylin and eosin (H&E) to evaluate the toxicity of the AISA system.

## Results

### Design of the AISA System

Although various molecular beacons based on toehold-mediated reactions or nanomaterial-mediated quenching have been created, simple conjugation with one unit of target miRNA to release one unit of the fluorescent molecule would not solve a key challenge in the *in vivo* detection of miRNA: the need to produce enough fluorescence to be captured by our charge-coupled device system despite their degradation in tissues. Thus, we urgently need to focus on finding a way to make the two double-stranded DNA molecules involving in the reaction and further amplify the fluorescence signal. We designed two double-stranded DNA that can react with the target miRNA to achieve this amplification effect. To achieve the effect of cyclic reaction, the second and third reaction must maintain a kind of seesaw thermodynamic equilibrium. The length of dangling end α in double-stranded SQ determines whether the reaction can occur spontaneously, while the length of dangling end β determines its ability to distinguish between targets and their analogs, which is defined as the discrimination factor *Q* (Zhang et al., 2012). Here, the discrimination factor *Q* can be calculated based on experiments.

$$\begin{array}{l} Q=\frac{\chi_{M}}{\chi_{S}} \end{array}$$

where χ_*M*_ is the signal produced by SQ in response to the target miRNA, M, and χ_*S*_ is the signal produced by SQ in response to the spurious analogs, S.

**Figure 1:**
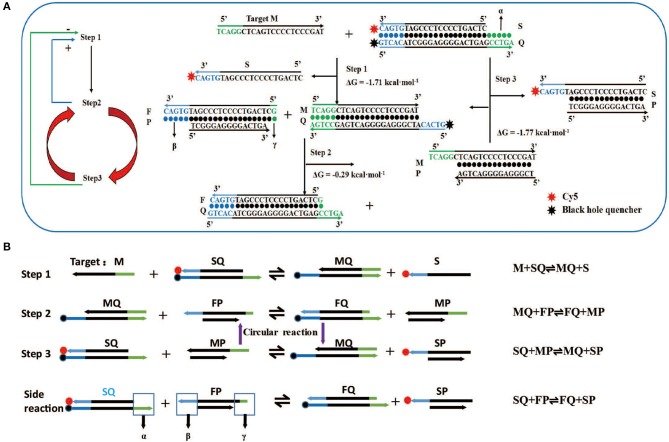
Design and reaction mechanism of artificial intelligent signal amplification (AISA) detection system. **(A)** Left panel: logical diagram of the three-step reaction, the second reaction and the third reaction occur in cycles, “+”: positive feedback, the second step reaction can promote the occurrence of the first step, “–”: negative feedback, the third step reaction has a negative feedback effect on the first step; right panel: schematic diagram of the reaction between two double-stranded DNAs (SQ and FP) in the artificial intelligence molecular amplification (AISA) system with target miRNA (e.g., Hsa-miR-484). The arrowheads point to the products and the reactants are on the other sides. The three reactions described in **(A)** correspond to the three reactions in **(B)**. **(B)** The step reaction diagram and reaction equation between AISA system and the target M.

The literature (Zhang and Winfree, 2009) indicates that the length of α must be ≥5 to ensure the occurrence of the first-step reaction, which is marked as design rule 1. In the second step, double-stranded MQ reacts with FP via dangling end β to produce stable double-stranded FQ and MP. Considering the above two reactions (Figure 1, steps 1 and 2), it is not difficult to find that dangling ends α and β correspond to each other, that is, the length of β influences the discrimination factor *Q* of the first step, and the length of α influences the discrimination factor of the second step. To ensure that the value of *Q* is acceptable, the length of dangling ends α and β should only be 5 or 6. Here, we take its length to be 5 (as design rule 2; also conforms to design rule 1), and later, we find that the length of β = 6 influences only the length of the strand *P*, so we will no longer discuss this value separately.

Different carriers have different delivery efficiency to minimize the effect of delivery efficiency on fluorescence intensity. In this study, Lipo3000 is favored as a carrier of biomaterials due to its high delivery efficiency and the ability to load DNA through the mutual attraction of positive and negative charges.

### The Reaction Principle and Optimization of the AISA System

For the first time, we successfully achieved *in vivo* visualization of miRNA and introduce a general design framework for the AISA system and explain the reaction principle (Figures 1A,B). The key innovation is the use of a second double-stranded DNA (FP) to initiate the next two-step cascade cyclic reactions due to the thermodynamics equilibrium based on the first-step reaction. In the process of the reaction, the target M is constantly substituted to participate in the cascade reaction. Under the condition of excessive SQ and FP, a sufficiently large signal was produced through these three-step reactions. For the first-step reaction, double-stranded SQ (Cy5 at the 3′ end of the S strand, BHQ2 at the 5′ end of the Q strand) reacts with the target miRNA (M) via dangling end α to generate single-stranded S carrying the signal molecule Cy5 and double-stranded MQ with a new dangling end, β. This reaction can be represented by the following reversible reaction equation: M + SQ ⇌ MQ + S. For the second-step reaction, double-stranded MQ reacts with FP via dangling end β to produce stable double-stranded FQ and unstable MP: MQ +FP ⇌ FQ + MP. This unstable MP easily reacts with SQ through its dangling end to generate MQ and SP, which can emit fluorescence: SQ + MP ⇌ MQ + SP (the third-step reaction). The reaction between the two components of the AISA system, SQ and FP, is undesirable, and the resulting fluorescence is considered noise. The side reaction is represented in the following equation: SQ + FP ⇌ FQ + SP (Figures S1A,B). The most interesting thing is that the product of the third step, MQ, is a reactant in the second step; meanwhile, the second- and third-step reactions maintain a seesaw equilibrium thermodynamically; thus, these steps can occur cyclically. The above reaction principle was verified in the following experiments (Figures S1E–H).

**Figure 2:**
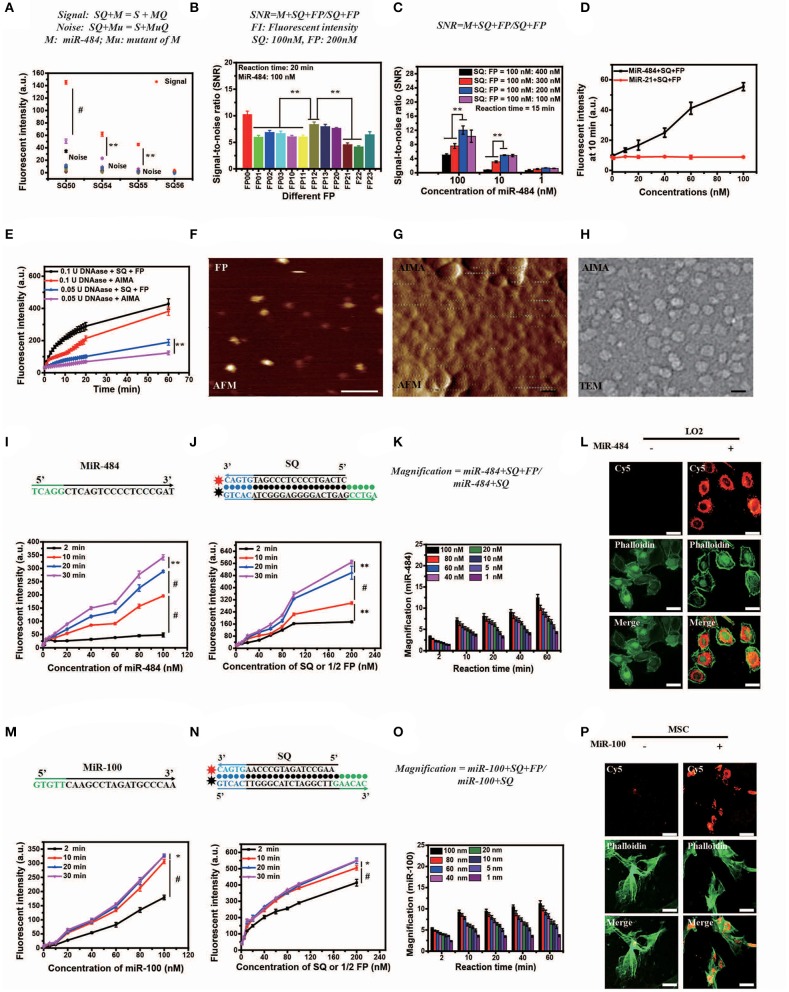
Optimization, characterization, and *in vitro* and intracellular dynamics of artificial intelligent signal amplification (AISA) systems with target RNA. **(A)** Fluorescence intensity generated by different SQs and miR-484 or its 11 mutants measured using a fluorospectrophotometer [signal indicates the fluorescence intensity generated by miR-484, noise indicates the fluorescence intensity generated by its 11 mutants (shown in Table S1)]. **(B)** Signal/noise ratio (SNR) generated by the reactions based on 12 kinds of double-stranded FP (the fluorescence intensity generated by SQ and FP with miR-484 was considered signal, while fluorescence intensity generated by SQ and FP without miR-484 was considered noise). **(C)** SNR generated by different ratios of double-stranded SQ/FP and miR-484 at 15 min after reaction (signal and noise are the same as in A). **(D)** Fluorescence intensity generated by miR-484 and miR-21 (SQ and FP is designed for detection of miR-484). **(E)** Fluorescence intensity generated by reactions between DNase (0.1 U, 0.05 U) and SQ, FP, or the AISA system. **(A–E)** Bars represent mean ± SD, *n* = 5. **(F–H)** From left to right is the atomic force microscopy (AFM) image of double-stranded FP and the AISA system, the transmission electron microscopy (TEM) image of the AISA system; scale bar, 50 nm. Throughout the paper, ANOVA and the *t*-test were used to analyze the means, ^*^p < 0.05, ^**^p < 0.01, ^#^*p* < 0.001. **(I)** Core sequence of miR-484; fluorescence intensity generated by the reaction between double-stranded SQ (100 nM), FP (200 nM), and different concentrations of miR-484. **(J)** Sequence of double-stranded SQ for detection of miR-484; fluorescence intensity generated by the reaction between miR-484 (100 nM) and different concentrations of double-stranded SQ and FP (SQ/FP = 1/2). **(K)** Magnification of the reaction between SQ (100 nM) and different concentrations of miR-484 with and without double-stranded FP. **(L)** Bright field microscopic image of LO2 cells transfected with or without miR-484. Fluorescence image of LO2 cells transfected with or without miR-484 after adding the AISA system. Fluorescence image of LO2 cells transfected with or without miR-484 stained with fluorescein isothiocyanate (FITC) -phalloidin. Merged image of LO2 cells transfected with or without miR-484. **(M)** Core sequence of miR-100; fluorescence intensity generated by the reaction between double-stranded SQ (100 nM), FP (200 nM), and different concentrations of miR-100. **(N)** Sequence of double-stranded SQ for detection of miR-100; fluorescence intensity generated by the reaction between miR-100 (100 nM) and different concentrations of double-stranded SQ and FP (SQ/FP = 1/2). **(O)** Magnification of the reaction between SQ (100 nM) and different concentrations of miR-100 with and without double-stranded FP. **(P)** Bright field microscopic image of mesenchymal stem cells (MSC) transfected with or without miR-100. Fluorescence image of MSC transfected with or without miR-100 after adding the AISA system. Fluorescence image of MSC transfected with or without miR-100 stained with FITC-phalloidin. Merged image of MSC transfected with or without miR-100. **(I–P)** Scale bars, 10 μm (ANOVA and the *t*-test were used to analyze the means, ^*^p < 0.05, ^#^*p* < 0.001).

The length of dangling end α determines whether the first-step reaction can occur spontaneously, while the length of dangling end β determines its ability to distinguish between targets and their analogs (Zhang et al., 2012). We designed 11 variants of miR-484 (miRNA usually has a high probability of point mutation at specific positions and insertion and deletion mutants in human body Table S1) and four different kinds of double-stranded SQ (Table S2) to verify the discrimination ability of SQ. Compared with others (SQ50, SQ54, and SQ56), SQ55 performed best and was ultimately selected as the first double-stranded DNA in our AISA system (**Figure 2A**). Notably, the length of the dangling end γ determines whether the above side reaction can occur [the side reaction refers to the reactions that occur between SQ and FP when there is no target RNA (see Figure 1B)], so we limited the length of γ to 4 or less. To further demonstrate the optimal structure of double-stranded FP, 12 double-stranded FP chains (listed in Table S3) were designed to participate in the experiments, and the SNR was tested with miR-484. SNR was defined as $SNR=\frac{FL_{m}}{FL_{s}+FL_{m}}$, where FL_m_ is the fluorescence intensity generated by the reaction among miR-484 with double-stranded SQ and FP and FL_s_ is the fluorescence intensity generated by the reaction between double-stranded SQ and FP. The results in Figure 2B show that the overall performance of FP00 is optimal in terms of the SNR. However, the fluorescence intensity derived by FP00 (137 a.u.) is lower than that of FP12 (152 a.u.) at the same conditions. Thus, to ensure the miRNA detection depth, FP12 was selected as the following application. We used four different SQ/FP ratios and measured the resulting SNR. Although higher FP concentrations increased the signal intensity, the noise cannot be ignored. From the comparison of the SNR of the four reactions with different concentrations, the ratio of SQ/FP = 1/2 was selected of our AISA system (Figure 2C).

### Characterization of the AISA System *in vitro*

In addition, to assess the specificity of the system, fluorescence intensity generated by miR-484 and miR-21 were detected. With the increase in RNA concentration, the fluorescence intensity generated by miR-484 was much higher than that generated by miR-21 (Figure 2D). The fluorescence intensity generated by miR-484 was about five times of that generated by miR-21 especially at high RNA concentrations. For transportation of the active components (SQ and FP) to the target to guarantee that the detection reaction will occur, a suitable carrier is also indispensable. Lipo3000 has been experimentally (Figure 2E) demonstrated to be able to resist the hydrolysis of DNase to some extent for 60 min, which may be due to the steric effect of DNase itself.

To better understand the *in viv*o detection principle, the appearance and microscopic behaviors of SQ and FP in the AISA system were observed using transmission electron microscopy (TEM) and high-speed atomic force microscopy (AFM), respectively (Kodera et al., 2010). TEM and AFM images of SQ/FP (Figures 2F–J; Movies 1,2) demonstrated that the length of the double-stranded DNA was ~10 nm, the width was ~2 nm, and the area was ~20 nm^2^. When double-stranded SQ and FP were loaded onto the surface of the liposome, an ~50 nm sphere formed. After adding the target, miR-484, the reaction between double-stranded SQ, FP, and the target was observed by AFM (Movie S3). In addition, to obtain high-purity double-stranded DNA, all synthetic products in this work were purified by high-performance liquid chromatography. The mass spectrometry data (Figures S1I–P) showed that the purity of the product we synthesized was >99%, which ensured that the noise caused by product impurity was <1%.

### Visualization of miR-484 *in vitro* and in Cells

Although *in vitro* DNA hybridization kinetics based on some sequences have led to a set of mature theories (Dirks and Pierce, 2003, 2004; Dirks et al., 2004, 2007; SantaLucia and Hicks, 2004; Zhang and Winfree, 2009; Zadeh et al., 2011; Zhang et al., 2012, 2018a; Wolfe and Pierce, 2015; Wolfe et al., 2017), a basic theory for quantifying miRNA *in vivo* is still lacking, which is restricted by technologies in other related fields, such as fluorescence reconstruction (Ntziachristos and Weissleder, 2001; Dirks and Pierce, 2003; Cong and Wang, 2005; Lasser et al., 2008; Zhang et al., 2015; Shibata et al., 2017), pharmacokinetics (Brownbill et al., 2018; Caro et al., 2018; Sharma et al., 2018), and others. To quantify miRNA *in vivo*, the *in vitro* reaction kinetics of SQ, FP, and the target miRNA must be accurately modeled mathematically. The *in vitro* experimental results demonstrated that the relationship between the fluorescence intensity generated by the reactions among SQ, FP, and miR-484 in 20 min, and the miR-484 concentration was not strictly linear and instead resembled a parabola (Figures 2I,J,M,N). Compared to a single double-stranded DNA that detects miRNA at the cellular level (Siuti et al., 2013; You et al., 2015, 2017; Ma et al., 2017), the AISA system consisting of two double-stranded DNAs (SQ and FP) can effectively amplify (more than five times within 10 min) the signals of interest in a synergistic manner and hence provide the possibility for *in vivo* visualization of miRNA without an external energy input (Figure 2K). The *in vitro* detection sensitivity of this pair of double-stranded DNAs was ~0.05 nM (reaction time = 10 min, concentrations of SQ = 10 nM, FP = 20 nM). The effect of temperature on the reaction was not significant at either 25 or 37°C (Figures S1C,D). Reducing the concentration of the reactants SQ and FP did not improve the detection sensitivity, which suggested that SQ and FP have a low probability of reacting in a short period of time and that the system formed with SQ and FP is relatively stable and resistant to internal noise.

To validate the reaction kinetics of the AISA system with a target miRNA in cells, we designed an experiment in which the AISA system reacted with miR-484 in LO2 cells (miR-484 was transfected into the LO2 cells). Dynamic confocal fluorescence images showed that, along with the AISA system, double-stranded SQ and FP were rapidly delivered into the cells in several seconds and reacted with intracellular miR-484 to emit fluorescence (Figure 2L; Figure S2A; Movie S4). After correction for the fluorescence quenching effect of the laser (Figure S2B), the fluorescence intensity was found to reach the maximum within 5 min, suggesting that the relatively limited space of the cytoplasm may accelerate the reaction and that the quenching effect must be considered when quantifying the fluorescence signal (Figure S2C).

### *In vivo* Visualization of Transfected and Induced miRNA Based on AISA System

**Figure 3:**
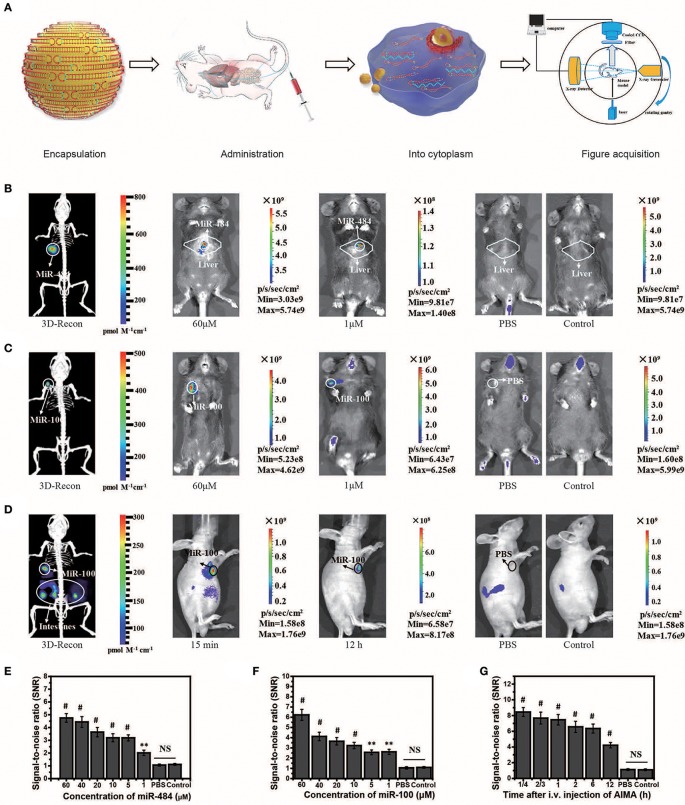
Visualization and 3D reconstruction of microRNA (miRNA) *in vivo* based on miR-484-injected and miR-100 injection mouse models. **(A)** Flow chart of the experiments, including encapsulation of double-stranded SQ and FP in liposomes; administration of the artificial intelligent signal amplification (AISA) system; process of double-stranded SQ and FP entering the cytoplasm; and image acquisition based on a dual-modality system including a high-sensitivity fluorescence system and a microcomputed tomography system. **(B)** Fluorescence images and 3D reconstructed image of mice injected with miR-484 (25 μl, 20 μM) in the liver (depth, 2.5 mm) at 10 min after i.v. injection of the AISA^484^ system. **(C)** Fluorescence images and 3D reconstructed image of mice injected with miR-100 in the femoral head at 10 min after i.v. injection of the AISA^100^ system. **(D)** Fluorescence images and 3D reconstructed image of SGC-7901-miR-100 tumor-bearing mice at 15 min after i.v. injection of the AISA^100^ system. **(E)** Signal/noise ratio (SNR) based on fluorescence images of different concentrations of miR-484-injected mice. **(F)** SNR in fluorescence images of mice injected with different concentrations of miR-100. **(G)** SNR in fluorescence images of SGC-7901-miR-100 tumor-bearing mice (ANOVA and the *t*-test were used to analyze the means, ^**^p < 0.01; ^#^*p* < 0.001; NS, no significant difference).

MiR-484 is an important biomarker expressed in the liver cells during the stage of transformation from liver cirrhosis to cancer (Yang et al., 2016b). To locate miRNA in the liver, we needed to reconstruct the 3D position of the light source based on the system (Figures 3A–D). Herein, we also experimentally validated the universality of the strategy using the AISA system to detect miR-484 in different animal models (miR-484-injected BALB/c mice in Figure S6, miR-484-injected C57 mice in Figure S7, and miR-484-injected rats in Figure S8). *In vivo* fluorescence images of the C57 mouse model demonstrated that miR-484 could be detected in the liver, and the 3D location of miRNA could be reconstructed based on our previous algorithms (Figure 3B). The above results indicated that fluorescence was generated by the reaction between miR-484 and the probe by comparing to the control group (Figures 3B,C). No fluorescence was produced without miR-484 within 30 min. Inevitably, with the extension of time, some fluorescence will be generated by the decomposition of probes such as the body's own enzymes (Figure S6E). Figure 3A shows the detection process and a schematic diagram of the dual-modality imaging system with microcomputed tomography. We can use this system to acquire enough data to reconstruct the 3D location of the internal miRNA.

To verify the ability of the AISA system to detect miRNA in a deep target, we established mouse models by injecting miR-484 at a depth of 4 mm in the liver on BALB/c mice (10 weeks, weight >25 g). The fluorescence images and 3D reconstruction results showed (Figure S3) that the AISA system also had good detection depth for targets at a depth of 4 mm. Two-photon fluorescence and microscopic images of a 60-μm slice also verified the detection capability of the AISA system.

In addition, to evaluate the ability of the AISA system to distinguish between a tumor and regions with high miR-484 expression, we established miR-484-injected and tumor-bearing mouse models by injecting 10 μl of miR-484 (100 μM; depth, 3 mm) into the liver of DEN-induced tumorigenic mice (Figure S4). The fluorescence images obtained with our system and microscope showed that the AISA system has the ability to distinguish the areas of high miR-484 expression from single or multiple tumors. The corresponding statistical data indicated that the SNR of the high miR-484 expression region to background (liver and other location) was >2. Similarly, we constructed rat models with five miR-484-injection foci to verify the horizontal spatial resolution of the AISA system. The fluorescence images demonstrated that when the distance between two of the miR-484-injected centers was more than 2 mm, the AISA system could distinguish them well (Figure S5).

### Scalability of the Framework for AISA System

Strikingly, to verify the scalability of the framework for AISA system, we successfully designed and synthesized AISA^100^ with minimal adjustment to detect miR-100 *in vitro* (Figures 2M–P) [an important molecular marker of the osteogenic differentiation of mesenchymal stem cells and also a biomarker associated with the progression of gastric cancer (Ueda et al., 2010; Frith et al., 2018)] and in the femoral head and in the tumor-bearing mice (Figures 3C,D,F,G). However, different miRNAs have different base sequences, and changing the base sequences of the components in the AISA detection system would influence the reaction kinetics, and a series of experiments must be performed to validate the design of the AISA system. From Figures 2I–M, we found that the fluorescence intensity increased depending on the concentration of miRNA. However, it can be also found from the curves at different reaction times that the two systems have different velocities at the initial stage of reaction. The response speed of the AISA^100^ system is much faster than that of the AISA^484^ system in 10 min (Figures 2G,N). Therefore, the magnification reached more than 10 times in the first 10 min, and the subsequent growth slowed down (Figures 2K,O).

The *in vivo* fluorescence images and their SNR demonstrated that miR-100 can be distinguished from surrounding tissues with a high SNR ratio and even at locations close to the bladder (Figure S6E). Statistical data of continuous observation experiments (Figure S6H) showed that the fluorescence intensity on the surface of the femoral head was the strongest at 60 min, which may be caused by the difference in the metabolic dynamics of the AISA^100^ system between the femoral head and the liver. Fluorescence images of SGC-7901-miR-100 tumor-bearing mice (miR-100 transfected into SGC-7901 gastric cancer cells) demonstrated that, until 12 h, SGC-7901-miR-100 can be visualized clearly because the formation of dense tumor tissue delays the metabolism of signal-stranded S and SP to some extent (Figure 3D).

In some *in vivo* experiments performed to visualize transfected miRNA, we found that the fluorescence in the intestinal tract of mice produced a relatively large amount of noise, so we designed a suppression experiment to verify the possible influencing factors. In general, fasting inhibited the secretion of relevant digestive enzymes, and the secretion of gastrointestinal hormones may lead to the degradation of double-stranded SQ in the AISA system (Figure S7). Analysis of abdominal fluorescence intensity showed that both fasting and somatostatin (gastrointestinal hormone secretion inhibitors) had significant inhibitory effects on fluorescence at the time points of 10 and 30 min, and the significant difference in the inhibition effect decreased with the time of administration.

### Practicability of the AISA System

**Figure 4:**
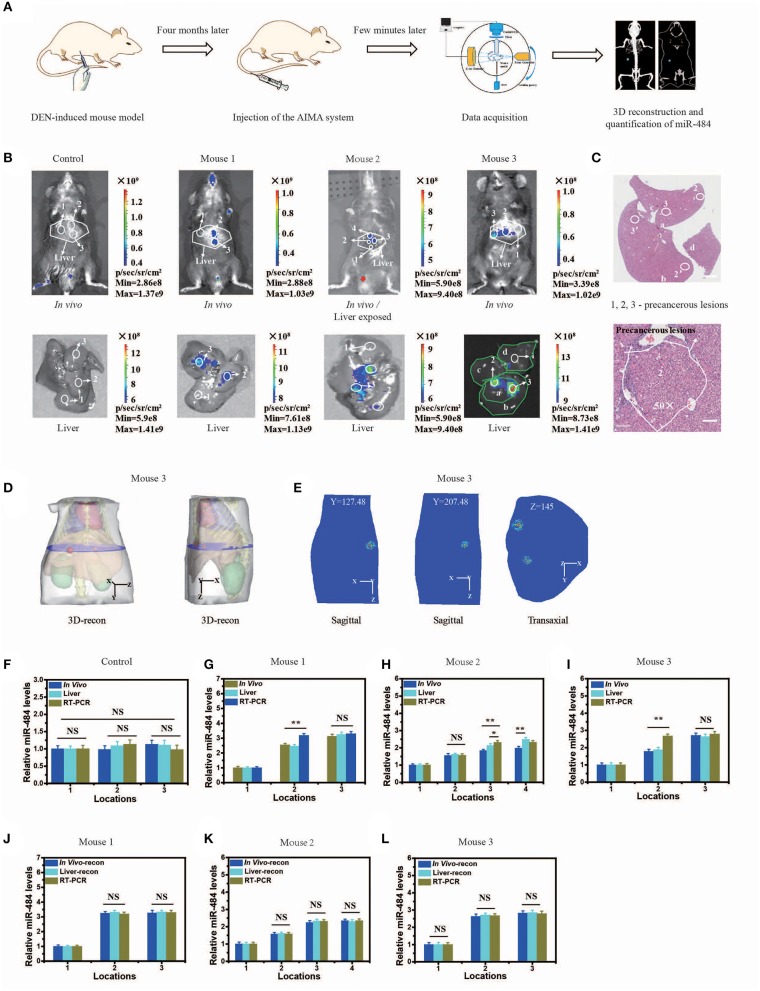
Visualization and three-dimensional (3D) reconstruction of miRNA *in vivo* based on diethyl nitrosamine (DEN)-induced mice and the microscopic image of liver slices with hematoxylin and eosin (H&E) staining. **(A)** Schematic illustration of the quantification of miR-484, including DEN-induced mouse model; injection of the artificial intelligent signal amplification (AISA) system (included SQ and FP); data acquisition; 3D reconstruction and quantification of miR-484. **(B)** Upper panel: fluorescence images of DEN-induced tumorigenic mice (for 4 months) and phosphate-buffered saline (PBS)-injected mice 15 min after i.v. injection of the AISA^484^ system (included SQ and FP). Lower panel: fluorescence images of livers from the above DEN-induced mice and PBS-injected mice. **(C)** Microscopic image of liver slices with H&E staining of the liver of mouse 3. The liver lobes are marked a–d, and the precancerous lesions are marked 2, 3, 2′, and 3′. Scale bars are 120 and 30 μm, respectively. **(D)** 3D reconstruction results of miR-484 location in mouse 3. **(E)** Two planes of the reconstructed miRNA location. **(F–I)** Relative miR-484 levels assessed by surface fluorescence intensity and real-time RT-PCR analysis of samples with a size <40 μm in 4-month-old PBS- or DEN-injected mice (mice 1–3). **(J–L)** Relative miR-484 levels assessed by reconstructed fluorescence intensity and real-time RT-PCR analysis of samples with a size <40 μm in DEN-injected mice (mice 1–3) (ANOVA and the *t*-test were used to analyze the means, ^*^p < 0.05; ^**^p < 0.01; NS, no significant difference).

To evaluate the practicability of the AISA system, we detected the expression of miR-484 with DEN-induced tumorigenic mouse models and human samples using our fluorescence imaging system, and the detection results were also validated with RT-PCR for all three mice and pathological H&E staining for mouse 3 (*in vivo Detection of MiR-484 with DEN-induced Mice and Human Samples*). Figure 4B proved again that no fluorescence was emitted from the liver without the presence of miR-484. Fluorescence images, 3D-reconstructed images, and their related statistical data (Figures 4B–L) showed that the amount of miR-484 in precancerous lesions was approximately twice that in a normal liver, and the conclusions obtained from *in vivo* fluorescence images and *ex vivo* liver images were basically consistent. In addition, the SNR of miR-484 detected by RT-PCR was also consistent with the results obtained by *ex vivo* fluorescence intensity statistics (Figures 4J–L); here, all the samples used in RT-PCR were dissected according to the depth information derived from 3D-reconstructed locations. Without reconstruction guiding sample selection, the deviations among three kinds of relative miR-484 levels were >18% (Figures 4G–I), and with reconstruction guidance, the deviations were <5% (Figures 4G–L). The deviation is calculated according to the following formula:

(1) $$\begin{array}{r} D=\frac{{\text{SNR}}_{\text{PCR}}-{\text{SNR}}_{\text{FI}}}{{\text{SNR}}_{\text{PCR}}}\times 100\% \end{array}$$

To demonstrate the accuracy of the location detected by the AISA system *in vivo*, the 3D location information was reconstructed, and the liver sections of mouse 3 were stained with H&E to observe the precancerous lesions (Figures 4C,D). We observed the formation of a precancerous lesion (Figure 4C), and the location of the precancerous lesion was highly consistent with the fluorescence location in *in vivo* and the *ex vivo* liver fluorescence images.

**Figure 5:**
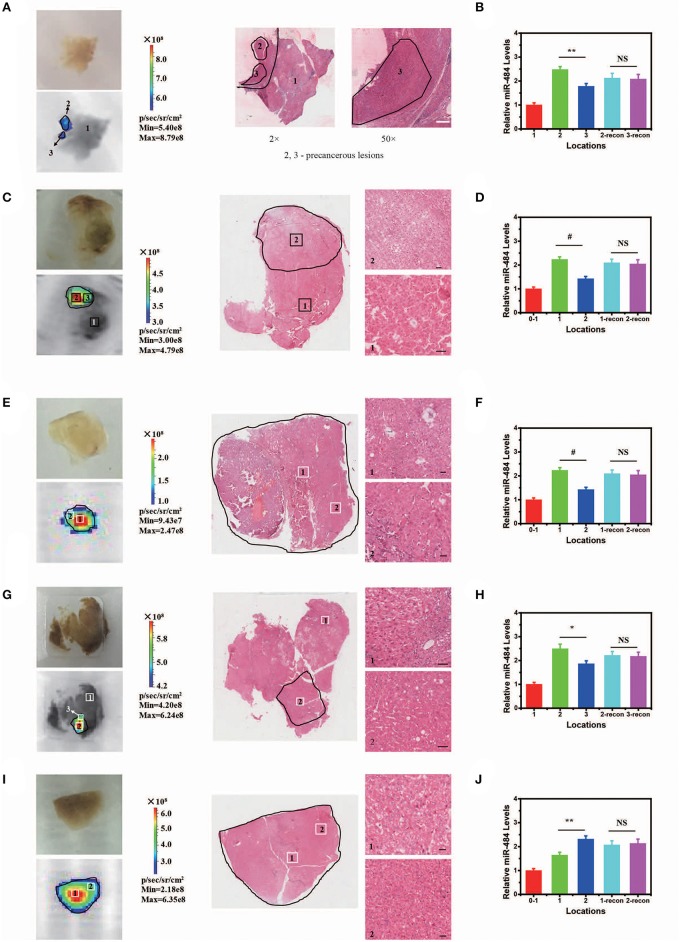
Visualization and three-dimensional (3D) reconstruction of miRNA *in vivo* Based on Human Liver Samples. **(A,C,E,G,I)** (from left to right) Bright field image and fluorescence image of human liver samples 1–5. Microscopic image of human liver samples 1–5. In **(A,C,G)**, normal tissue and precancerous lesions are marked 1, 2, and 3. Scale bars, 90 μm. In **(E,I)**, precancerous lesions are marked 1 and 2 according to the difference in fluorescence intensity. Scale bars, 60 μm. **(B,D,F,H,J)** Relative miR-484 levels at different locations in human liver samples 1–5 (ANOVA and the *t*-test were used to analyze the means, #*p* < 0.001; ^*^p < 0.05; ^**^p < 0.01; NS, no significant difference).

In addition, our AISA system was used to detect the precancerous lesions in five human liver samples from the Eastern Hepatobiliary Surgery Institute (Shanghai, China). The fluorescence images showed that our system was able to detect the region of high miR-484 expression in a short time (Figure 5) (~5 min after spraying the AISA system on the surface of the samples). The results were also verified by microscopic images of the liver sections with H&E staining, in which the boundaries of precancerous lesions (high-grade dysplastic nodule) were drawn by professional pathologists (Yang et al., 2016b). To assess the similarity of precancerous lesion locations obtained by the two methods, the boundaries were outlined, and the Dice coefficients were calculated. The results (Table S4) demonstrated that Dice coefficient for all five samples were more than 0.94, which indicated that our AISA system can accurately detect the boundaries of precancerous lesions. This excellent quality indicates that it has great application potential. For example, for liver transplantation patients, the subsequent treatment strategy can be determined according to whether the dissected liver has precancerous foci.

It is worth mentioning that low toxicity is needed to make the AISA system suitable for use, so we also measured the *in vivo* clearance speed of the AISA system and its effect on various organs, including liver, muscle, heart, spleen, kidney, stomach, lung, brain, and skin (Figures S8, S9). The fluorescence images and statistical data based on BALB/c mice showed that the AISA system could be almost completely cleared from the body within 12 h (Figure S8). A cell viability assay Figure S9A), serum test results (Figure S9B), and histopathological microscopic images (Figure S9C) all showed that the AISA system had no significant toxicity in animals (*Toxicity of the AISA System*).

## Discussion

The important role of miRNA in the physiological and disease development process is well known, but the *in vivo* visualization study of RNA is still almost blank. Although many strategies have been designed for the amplification of signal derived from miRNA (Yurke et al., 2000; Seelig et al., 2006; You et al., 2015, 2017; Ma et al., 2017; Fu et al., 2018), most of them amplified the miRNA signal *in vitro*. The *in vivo* visualization of miRNA will be a milestone event in the history of RNA detection, which will herald a new era of preventive medicine. However, the prerequisite is that signal derived from miRNA can be amplified *in vivo* first. The ability to design an artificial intelligent signal amplification system suitable for *in vivo* visualization of miRNA is not only interesting and significant on a fundamental basis but also offers key conceptual advantages in scalability over the current paradigm for early tumor diagnosis. In contrast to the situation for previous molecular beacon design efforts, the key design challenge in the current study was to obtain as many useful signals as possible while minimizing the introduction of noise. This seemingly contradictory demand imposes strict conditions on the dangling end of the two double-stranded DNAs to use chemical thermodynamics to promote the reaction and maintain the seesaw-type equilibrium (for the second and third reactions). Meanwhile, it also makes the reaction dynamics more regular, enabling it possible to simulate the process using mathematical models.

In addition, the experiments on human liver samples also demonstrate the practicability of the strategy, which cannot only be used for *in vitro* and *in vivo* detection of miRNA but also can be used for the evaluation of tumor prognosis and rapid intraoperative identification of tumor margins based on tissue samples. The importance of miRNA in life science applications, as well as the practicability and universality of the principles underlying this system, will support further applications in many fields, including disease progression assessment, stem cell tracing, and disease prognosis evaluation.

Still, there are many problems with AISA system that need to be addressed. According to the restriction of dangle end α in design rule 1 (α = 5), a total of 4^5^ different AISA system can be used to detect a target RNA. In addition, considering the limitation of dangle end β on design rule 2, more different AISA system can also be used for the visualization of miRNA. How to select the most suitable system for subsequent *in vivo* visualization and quantitative research is an urgent problem to be solved in the future studies. For the mechanism research about miRNA-related diseases, the *in vivo* quantification is also imminent. This is limited by advances in a variety of technologies including fluorescence position reconstruction, fluorescence photon quantification, reaction dynamics modeling of miRNA, and so on. All in all, based on these advances, we will be able to calculate the concentration of RNA in the body in real time in the future.

## Data Availability Statement

All datasets generated for this study are included in the article/Supplementary Material.

## Ethics Statement

The animal study was reviewed and approved by the Institutional Animal Care and Use Committee at Peking University and the Eastern Hepatobiliary Surgery Hospital Research Ethics Committee.

## Author Contributions

XM wrote the manuscript in addition to designing the AISA system, performing, analyzing all experiments, and build the quantitative framework. XM and KZ performing the *in vitro* kinetics experiments. XM, LC, WZ, YY, BZ, KZ, and SZ performing the animal experiments. PW and ML assisted the cell experiments. YG assisted with the imaging acquisition experiments. XM, LZ, and ZS analyzing the data and constructed the framework for *in vivo* quantification of ncRNA. HW assisted with experiment design, manuscript preparation, and data/image analysis. XM and JT designed, supervised, analyzed all experiments, and in addition to assisting with manuscript preparation.

### Conflict of Interest

A patent application has been submitted based in part on results presented in this manuscript. XM and JT are listed as the inventors. The remaining authors declare that the research was conducted in the absence of any commercial or financial relationships that could be construed as a potential conflict of interest.
