# Supplementary material for: An Artificial Intelligent Signal Amplification System for in vivo Detection of miRNA
Source: Front Bioeng Biotechnol. 2019 Nov 21;7:330. doi: 10.3389/fbioe.2019.00330 (PMC6882290; doi:10.3389/fbioe.2019.00330)
Supplement: Supplementary file 5 [file Data_Sheet_1.PDF]

Supplementary Information for

An Artificial Intelligent Signal Amplification System for *in vivo* Detection of Non-coding RNA

Xibo Ma[1,2*], Lei Chen[3], Yingcheng Yang[3], Weiqi Zhang[2,4], Kun Zhang[1,5], Bo Zheng[3], Lin Zhu[1,2], Zheng Sun[1,2], Shuai Zhang[1,2], Yingkun Guo[5], Hongyang Wang[3*], Jie Tian[1,2,6*]

[1]CAS Key Laboratory of Molecular Imaging, Institute of Automation, Chinese Academy of Sciences, Beijing 100190, China.
[2]The University of Chinese Academy of Sciences, Beijing, 100049, China.
[3]International Co-operation Laboratory on Signal Transduction, Eastern Hepatobiliary Surgery Institute, Second Military Medical University, Shanghai 200438, China.
[4]National Laboratory of Biomacromolecules, CAS Center for Excellence in Biomacromolecules, Institute of Biophysics, Chinese Academy of Sciences, Beijing, China.
[5]Department of Radiology, West China Second University Hospital, Sichuan University, Chengdu, Sichuan, P.R. China.
[6]Beijing Advanced Innovation Center for Big Data-Based Precision Medicine, Beihang University, Beijing 100191, China.

Corresponding author: Xibo Ma, Hongyang Wang, Jie Tian
Email: xibo.ma@ia.ac.cn, hywangk@vip.sina.com, jie.tian@ia.ac.cn

This PDF file includes:

Figs. S1 to S9
Tables S1 to S4
Captions for movies S1 to S4

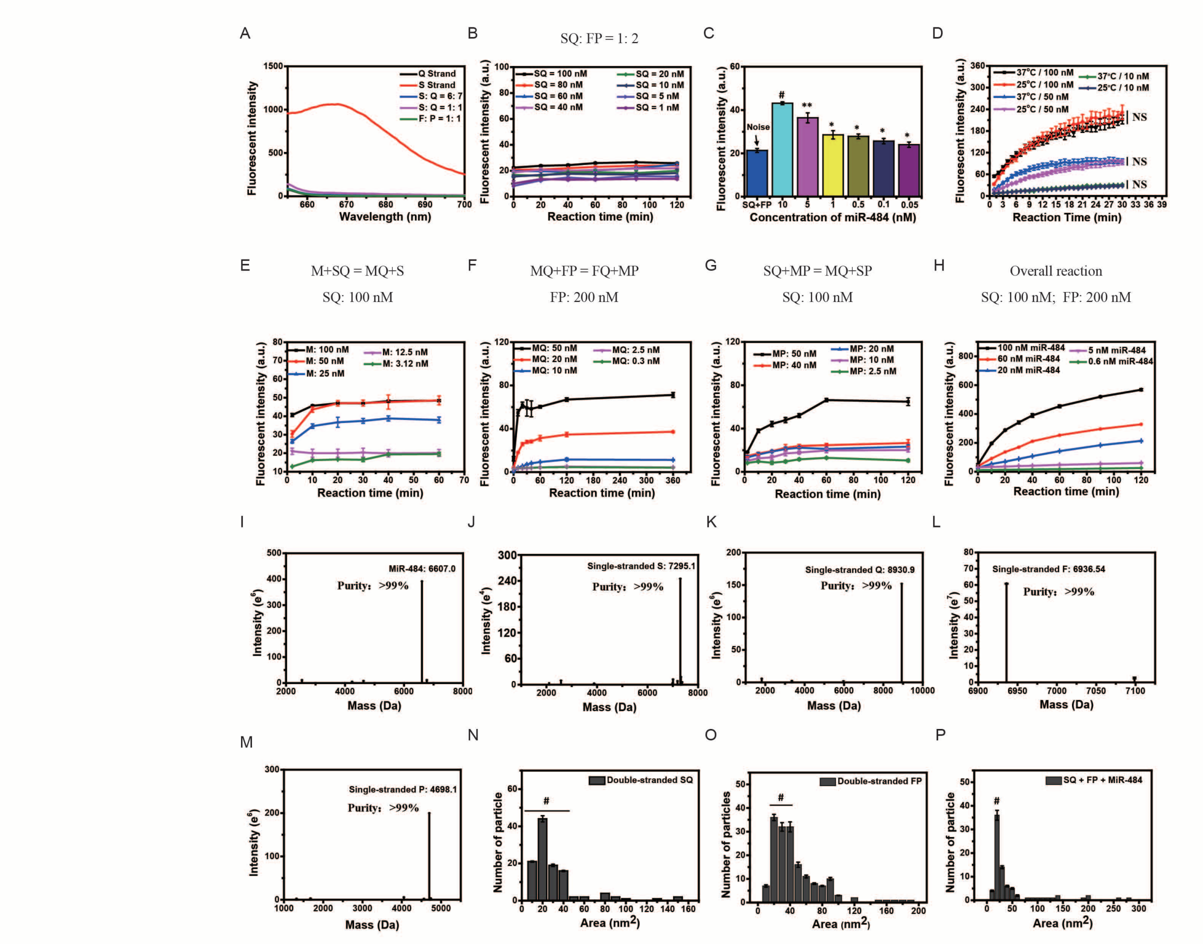

FIGURE S1| Characteristics of AIMA system. (A) Fluorescence intensity of single-stranded S, single-stranded Q, double-stranded SQ, and double-stranded FP. (B) Fluorescence intensity generated by the reaction between double-stranded SQ and FP without miR-484 (internal noise). (C) Fluorescence intensity of reactions among SQ (10 nM), FP (20 nM) and miR-484 (10 nM, 5 nM, 1, 0.5 nM, 0.1 nM, 0.05 nM). (D) Fluorescence intensity of reactions among SQ (100 nM), FP (200 nM) and different concentrations of miR-484 (100 nM, 50 nM, 10 nM) in 30 min at 25°C or 37°C (ANOVA and the t-test were used to analyse the means, $*p < 0.05$, $**p < 0.01$, $\#p < 0.001$, NS, no significant difference). (E-H) The fluorescence produced by each step of the reaction (E-G) and the overall reaction(H) (I-M) Mass spectra by stir-bar sorptive extraction and liquid-desorption-LC/MS negative mode analysis (SBSE-LD-LC/APCI-MS) of every strand and AFM-based DNA strand area statistics. (I) Single-stranded miR-484.(J)Single-stranded S. (K)Single-stranded Q. (L) Single-stranded F. (M) Single-stranded P. (N)Number of double-stranded SQ molecules in different areas. (O) Number of double-stranded FPs in different areas. (P)Number of mixtures (SQ/FP/miR-484) in different areas (ANOVA and the t-test were used to analyse the means, $\#p < 0.001$).

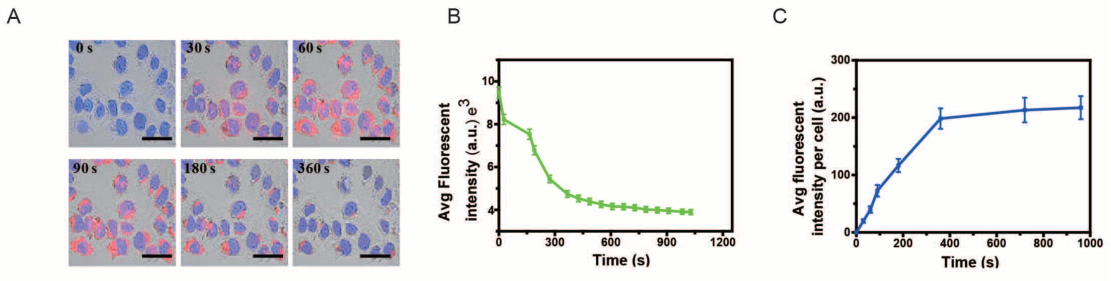

FIGURE S2| The intracellular kinetics of AIMA system reacting with miR-484 (A) Fluorescence image of LO2-miR-484 at different times after adding the AIMA system (SQ = 100 nM, FP = 200 nM); measurements were performed using LSCM. Scale bars, 20μm. (B) Fluorescence intensity decay of the signal strand (1 μM) measured using dynamic laser scanning confocal microscopy (LSCM) (UltraVIEW Vox, Germany) with a 50 mW laser at different times.(C)Average fluorescence intensity per cell (a. u.) determined using ImageJ (relative fluorescence intensity, the high is set as 200) after compensation..

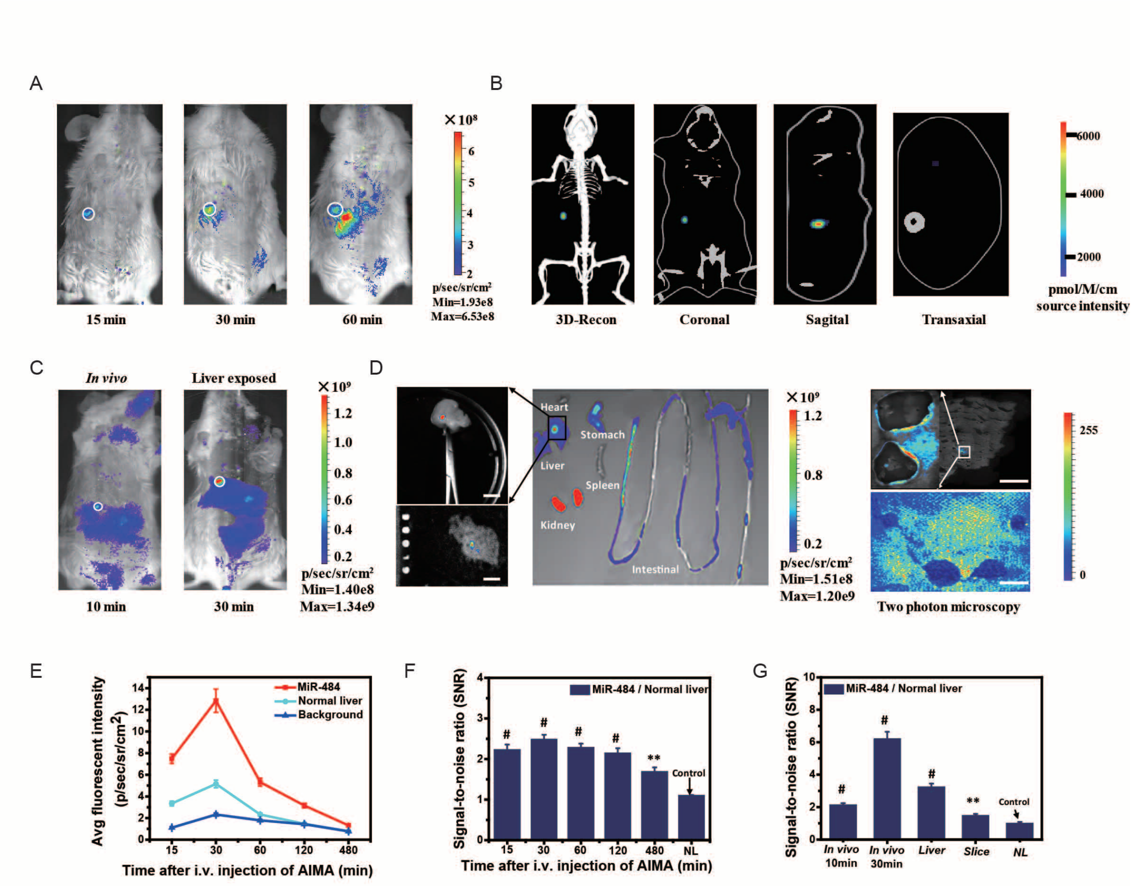

FIGURE S3| *In vivo* visualization of miR-484 with 4mm depth. (A) *In vivo* fluorescence image of a miR-484-injected BALB/c mouse at 15 min, 30 min, and 60 min after administration of the $AIMA^{484}$ system (miR-484 was injected in the mouse liver at an injection depth of 4 mm). (B) 3D reconstruction results of miR-484-injected mice 15 min after administration of the $AIMA^{484}$ system. (C) *In vivo* fluorescence image of a miR-484-injected BALB/c mouse at 10 min and 30 min (the liver exposed) after administration of the $AIMA^{484}$ system. (D) Left: Fluorescence image of the liver (upper panel) and a liver slice taken from a pathology section (60 µm) (lower panel). Scale bars, 1cm. Middle: Fluorescence image of different organs (heart, stomach, liver, spleen, kidney, intestinal) extracted from the mouse at 30 min after i.v. injection of the $AIMA^{484}$ system. Right: Microscopy image of the above a liver slice (4 ×) and partially enlarged detail (left panel, 40×) (upper panel) and two-photon microscopy image of the above slice (lower panel). Scale bars, 1 mm (upper panel), 60 µm (lower panel). (E) Average fluorescence intensity of the first mouse in Fig. S6A at different times (15 min, 30 min, 60 min, 2 h, 8 h). (F) SNR of miR-484 in Fig. S6A. (G) SNR of miR-484 of liver and section slice in Extended Fig. 6c. (ANOVA and the t-test were used to analyse the means, $**p < 0.01$, $\#p < 0.001$).

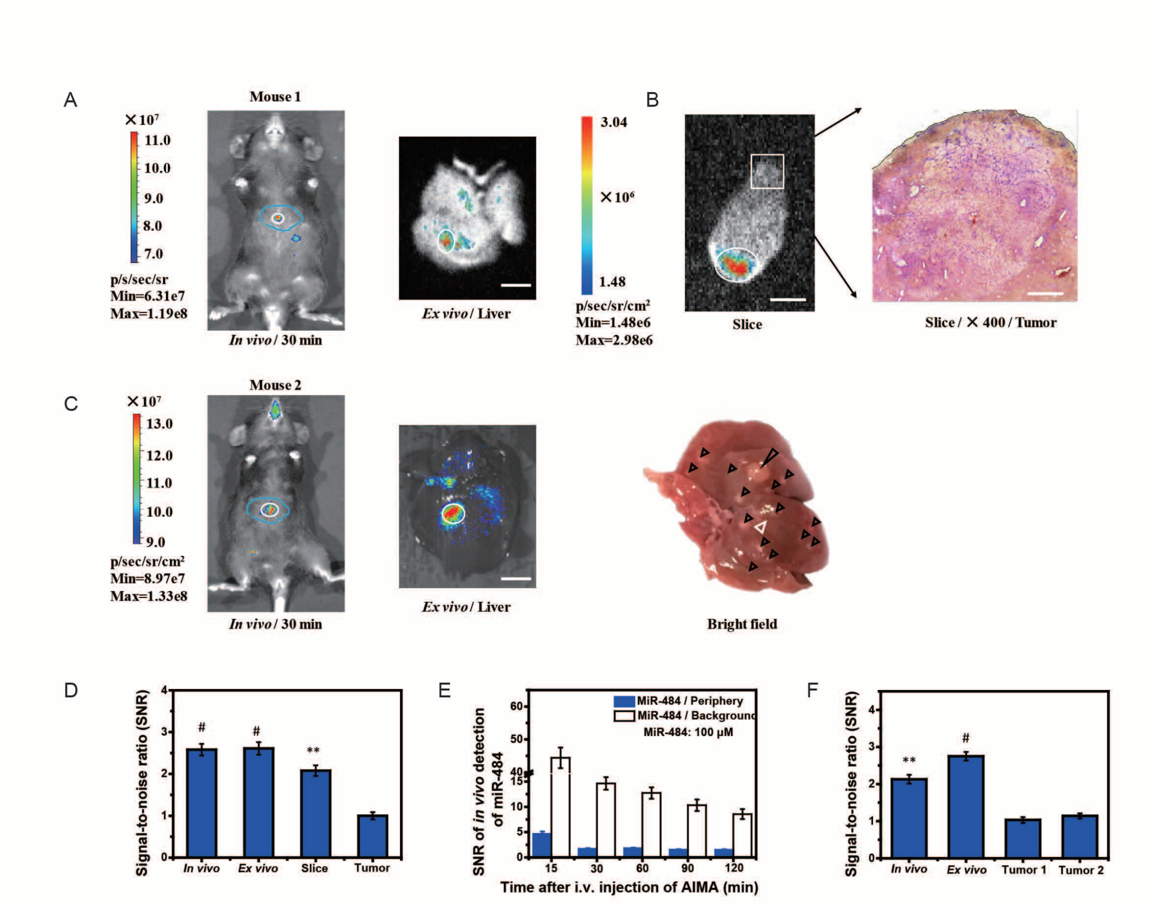

FIGURE S4| ***In vivo*** **visualization of miR-484 based on tumor-bearing mice.** (A) Fluorescence images of miR-484-injected tumour-bearing mice (25 μL of miR-484 (40 μM) was injected into C57BL/6N mice 5 months after intraperitoneal administration of DEN). The right image is a fluorescence image of the liver. Scale bar, 1 cm. (B) Fluorescence image of a mouse liver slice and microscopy image of a mouse liver slice at 400× magnification. Scale bars, 3 mm (left), 100 μm (right). (C) Left: fluorescence image of miR-484 (10 μL of miR-484 (100 μM) was injected into C57BL/6N) mice at 30 min after i. v. injection of the AIMA system. The mouse was treated with DEN for 7 months. Middle: fluorescence image of mouse liver after sacrifice at 2 h. Right: bright field image of the mouse liver. The black triangle indicates the tumour, and the white triangle indicates the location of miR-484 in the liver. Scale bar, 1 cm. (D) SNR of the miR-484 relative to the periphery under different conditions (*in vivo, ex vivo,* mouse liver slice). (E) SNR of *in vivo* detection of miR-484 relative to the periphery or background of mice at different times after i.v. injection of the AIMA system. (F) SNR of miR-484 relative to the periphery under different conditions (*in vivo*, *ex vivo*). (ANOVA and the t-test were used to analyse the means, $**p < 0.01$, $\#p < 0.001$).

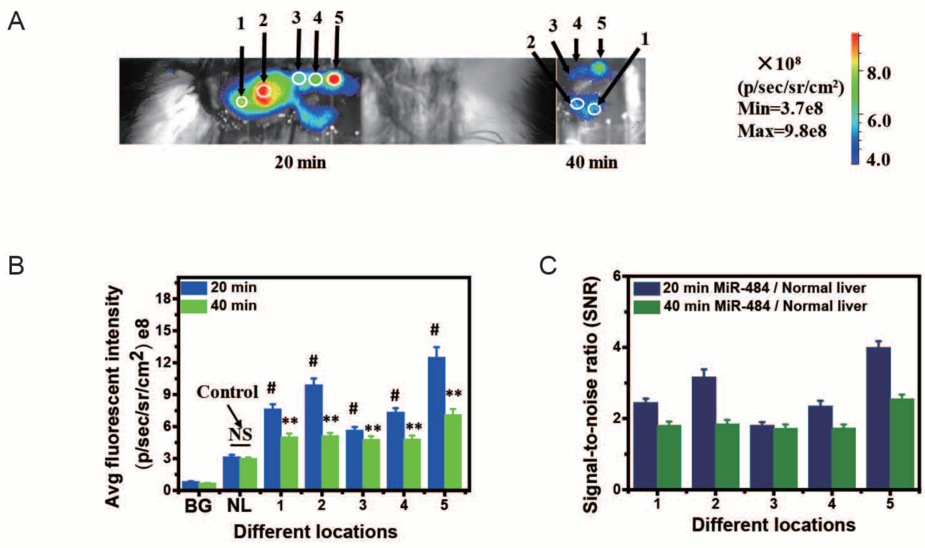

FIGURE S5| ***In vivo*** **visualization of multiple miR-484-injected rat model.** (A) Fluorescence image of a miR-484-injected rat (injection depth of miR-484 is 4 mm) at 20 min or 40 min after i. v. injection of the $AIMA^{484}$ system. For the volumes of 1, 3, and 4-10 µL of 100 µM miR-484; 2-15 µL of 100 µM miR-484; and 5-25 µL of 100 µM miR-484, the distance between 1 and 2 is approximately 4 mm, and the distance between 3 and 4 or 4 and 5 is approximately 2 mm. (B) Average fluorescence intensity for different miR-484 injection locations at 20 min and 40 min after i. v. injection of the $AIMA^{484}$ system. (C) SNR of miR-484 relative to the normal liver at different locations at 20 min and 40 min after i. v. injection of the $AIMA^{484}$ system (ANOVA and the t-test were used to analyse the means, $**p < 0.01$, $\#p < 0.001$).

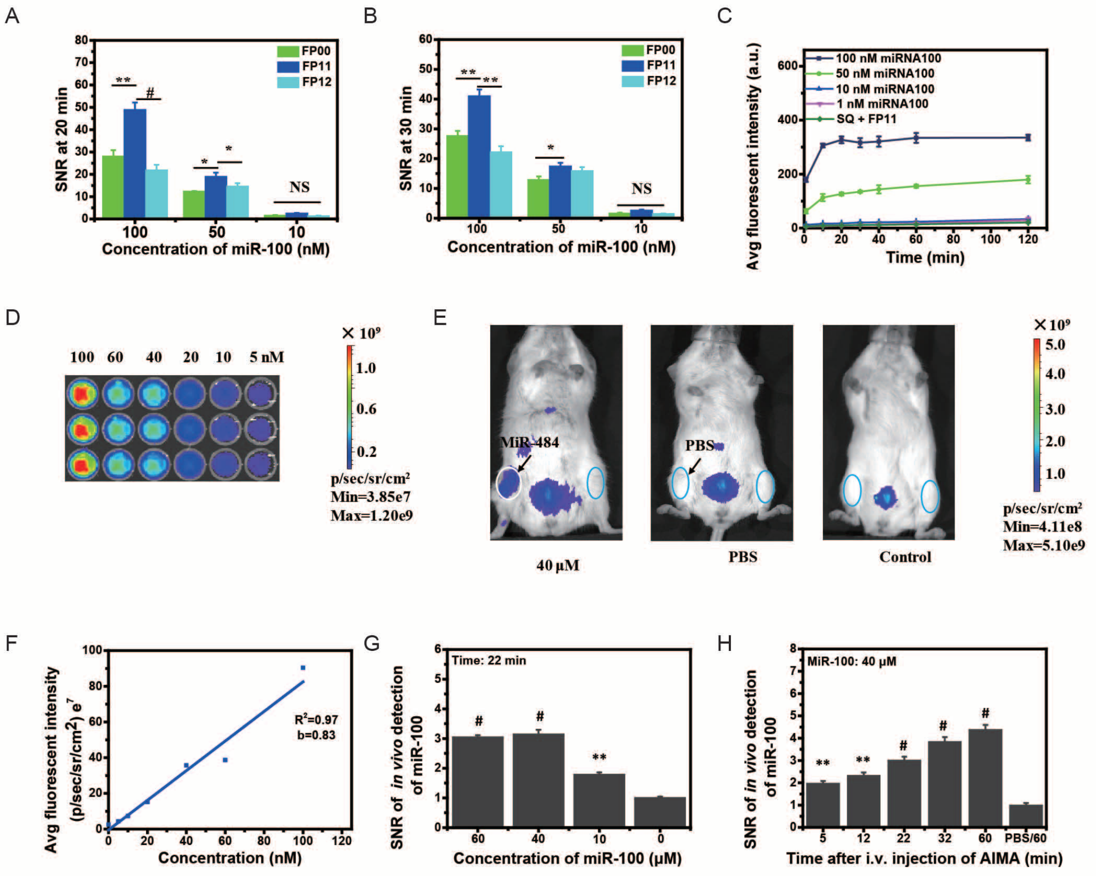

FIGURE S6| Detection of miR-100 *in vitro* and *in vivo*. (A) SNR for the reaction among SQ (100 nM), different types of FP (200 nM) and different concentrations of miR-100 at 20 min. (B) SNR for the reaction among SQ (100 nM), different FP (200 nM) and different concentrations of miR-100 at 30 min. (C) Average fluorescence intensity generated by the reaction among double-stranded SQ (100 nM) and FP11 (200 nM) and different concentrations of miR-100 or SQ and FP without miR-100. (D) Fluorescence image of 300 µL of 100 nM single-stranded S in the AIMA^{100} system. (E) Fluorescence image of miR-100 bearing mice at different times after i.v. injection of the AIMA^{100} system (mice were treated with 25 µL of 40 µM miR-100, PBS for PBS group or nothing for control group in the femoral head of the left lower limb respectively). (F) Correlation between fluorescence intensity and the concentration of single-stranded S in the AIMA^{100} system (corresponding to Fig. S9D). (G) SNR of the *in vivo* detection of the miR-100 (25 µL miR-100 (60 µM, 40 µM, and 10 µM) was injected into the femoral head of Balb/C mice at a 2 mm depth); signal: the average fluorescence intensity of the target miR-100 (the region of the circle); noise: the average fluorescence intensity of other region around the signal. (H) SNR of miR-100-injected mice at different time after i.v. injection of the AIMA^{100} system (ANOVA and the t-test were used to analyse the means, $**p < 0.01$, $\#p < 0.001$).

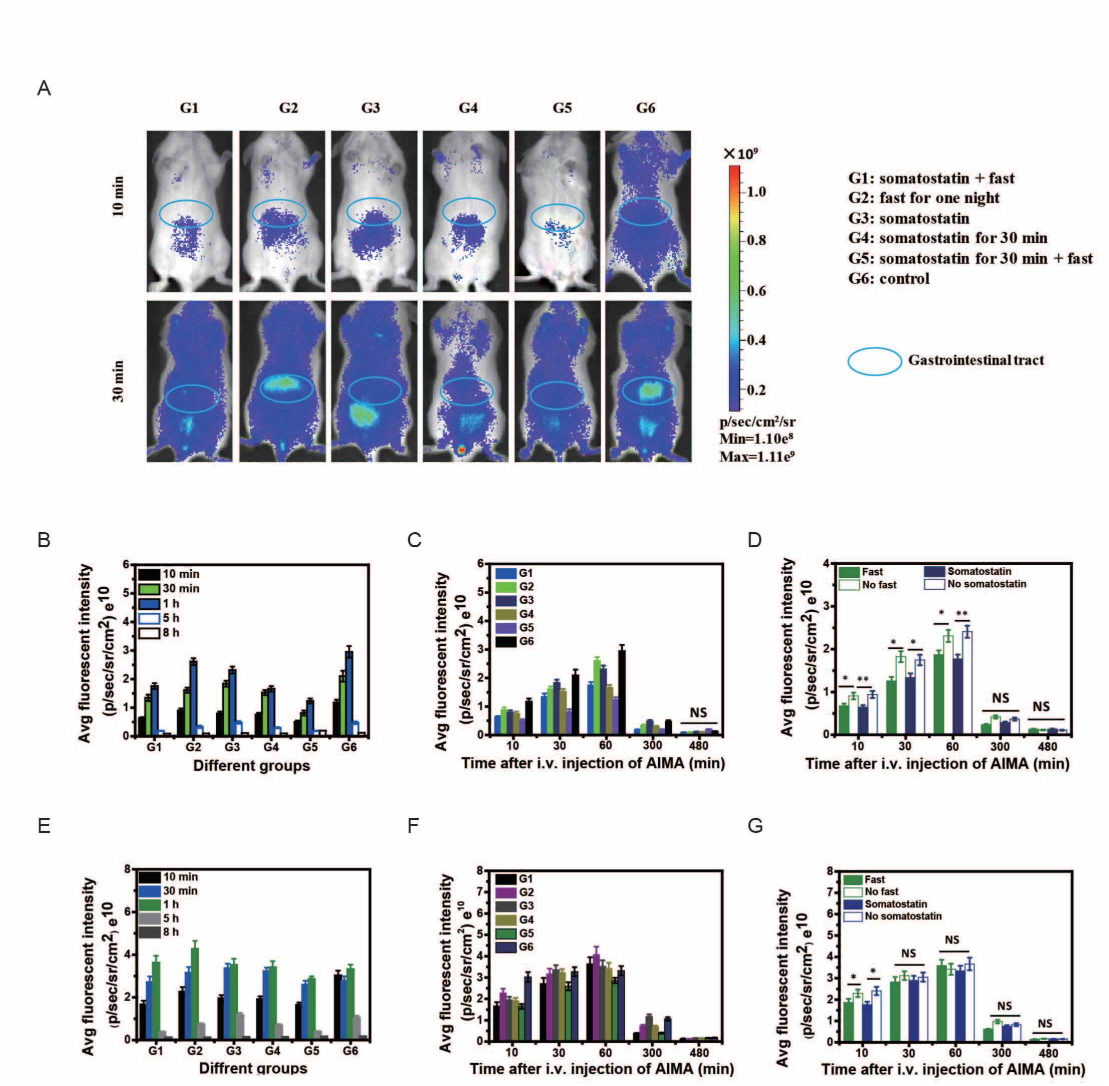

FIGURE S7| Effects of somatostatin and fasting on the AIMA system. (A) Fluorescence images of mice in different groups at 10 min and 30 min after i.v. injection of the AIMA system. G1: mice were treated with somatostatin before the AIMA system administration and fasting for one night. G2: mice were fasted for one night before the AIMA system administration. G3: mice were treated with somatostatin before the AIMA system administration. G4: mice were treated with somatostatin half an hour before the AIMA system administration. G5: mice were treated with somatostatin half an hour before the AIMA system administration and fasted for one night. G6: control group. (B) and (C) Average fluorescence intensity on mouse abdomen in different groups at different times after i. v. injection of the AIMA system. (D) Average fluorescence intensity of mouse abdomen with or without fasting and with or without somatostatin at different times after i.v. injection of the AIMA system. (E) and (F) Average fluorescence intensity of mice in different groups at 10 min, 30 min, 1 h, 5 h and 8 h after i. v. injection of the AIMA system. (G) Average fluorescence intensity of mice with or without fasting and with or without somatostatin at different times after i. v. injection of the AIMA system (ANOVA and the t-test were used to analyse the means, *$p < 0.05$, **$p < 0.01$, NS, no significant difference).

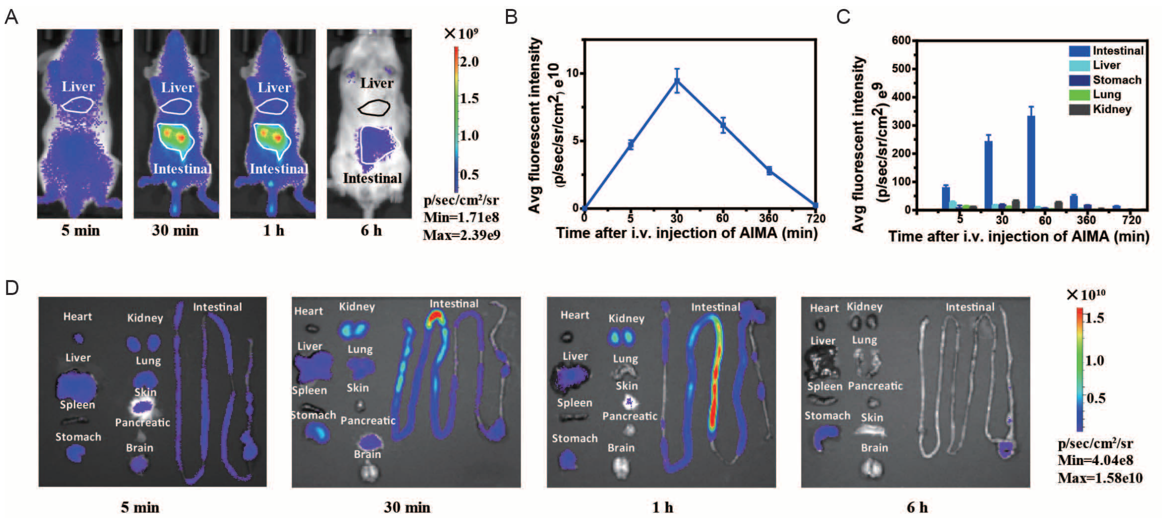

FIGURE S8| The distribution of AIMA system in different organs of the body at different times. (A) Fluorescence images of Balb/c mice at different times after i.v. injection of the AIMA system (the quencher strand was not linked to BHQ2). (B) The total average fluorescence intensity of mice at different times until 720 min after i.v. injection of the AIMA system. (C) The total average fluorescence intensity of major organs, including the intestines, liver, stomach, lung, kidney, at different times after i.v. injection of the AIMA system (ANOVA and the t-test were used to analyse the means). (D) Fluorescence images of different organs, including heart, kidney, liver, lung, spleen, pancreatic, stomach, skin, brain and intestines, dissected from mice at 5 min, 30 min, 1 h, and 6 h after i. v. injection of the AIMA system.

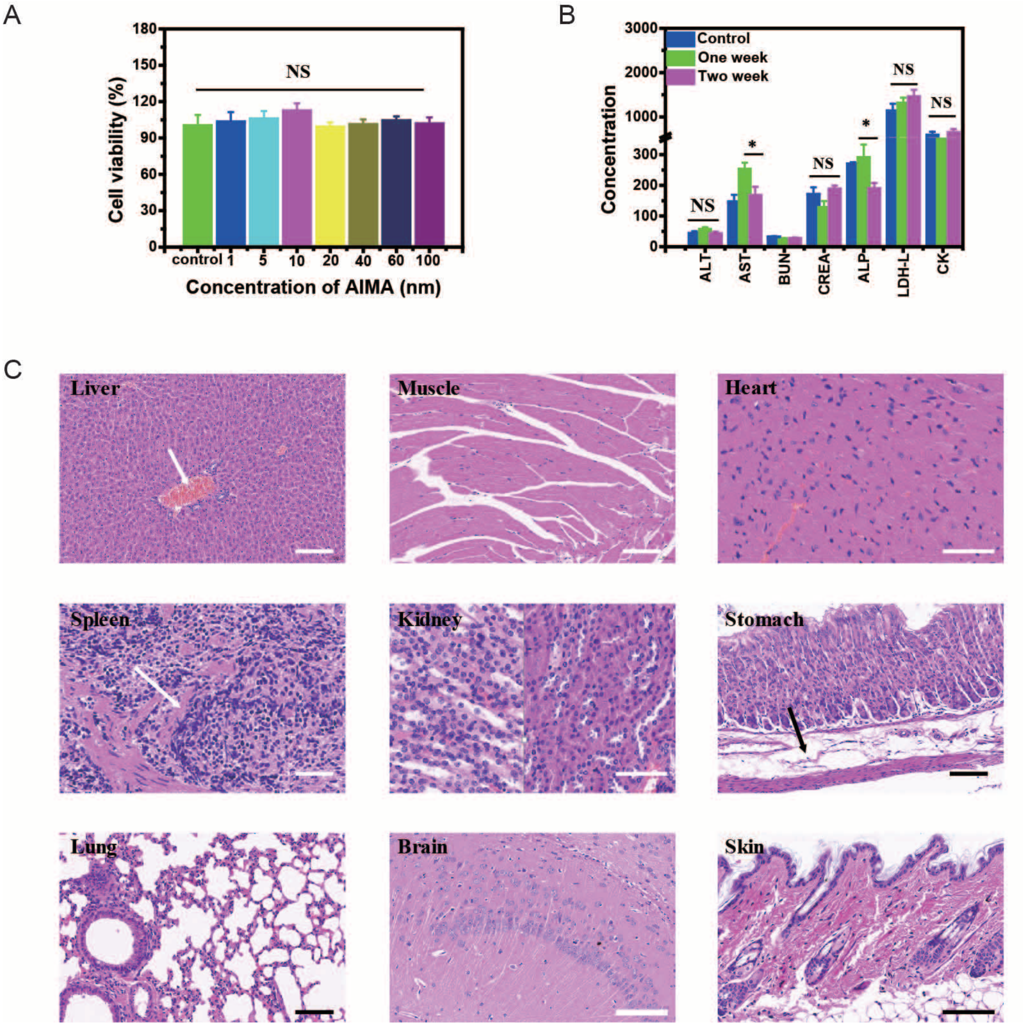

FIGURE S9| Toxicity of the AIMA system. (A) The viability of LO2 cells after incubation for 24 h with the AIMA system. (B) Concentrations of various indicators detected in serum, including creatine kinase (CK), lactate dehydrogenase (LDH), aspartate transaminase (AST), alanine aminotransferase (ALT), alkaline phosphatase (ALP), blood urea nitrogen (BUN), and creatinine (CREA). (C) Microscopic images of H&E-stained samples from various organs of mice 2 weeks after i. v. injection of the AIMA system (the results for the control group are not shown here), including the liver (a small amount of thrombus is visible in the portal vein in two samples), muscle, heart, spleen (Oat-like hyperplasia can be seen in the local splenic region in two samples), kidney, stomach (local connective tissue hyperplasia was found in a stomach tissue sample of one mouse), lung, brain and skin, Scale bars, 60 μm (ANOVA and the t-test were used to analyse the means, $*p < 0.05$, NS, no significant difference, n = 7).

TABLE S1| Sequence of 11 miR-484 variants.

Strand	Sequence	Number of bases
m1A	ACAGGCTCAGTCCCCTCCCGAT	22
m6G	TCAGGGTCAGTCCCCTCCCGAT	22
m11A	TCAGGCTCAGACCCCTCCCGAT	22
m11G	TCAGGCTCAGGCCCCTCCCGAT	22
m11C	TCAGGCTCAGCCCCCTCCCGAT	22
d11	TCAGGCTCAGCCCCTCCCGAT	21
i11A	TCAGGCTCAGATCCCCTCCCGAT	23
i11T	TCAGGCTCAGTTCCCCTCCCGAT	23
i11G	TCAGGCTCAGGTCCCCTCCCGAT	23
i11C	TCAGGCTCAGCTCCCCTCCCGAT	23
m16A	TCAGGCTCAGTCCCCACCCGAT	22

TABLE S2| Sequence of different double-stranded SQ molecules for detection of miR-484 and their 5’ and 3’ functional groups.

Strand	Sequence	5’ Functionalization	3’ Functionalization
Quencher-55	GTCACATCGGGAGGGGACTGAGCCTGA	5'BHQ2	
Signal-55	CTCAGTCCCCTCCCGATGTGAC		3`Cy5
Quencher-54	TCACATCGGGAGGGGACTGAGCCTGA	5`BHQ2	
Signal-54	CTCAGTCCCCTCCCGATGTGA		3`Cy5
Quencher-56	CGTCACATCGGGAGGGGACTGAGCCTGA	5`BHQ2	
Signal-56	CTCAGTCCCCTCCCGATGTGACG		3`Cy5
Quencher-50	ATCGGGAGGGGACTGAGCCTGA	5`BHQ2	
Signal-50	CTCAGTCCCCTCCCGAT		3`Cy5

Table S3| Sequence of 12 different double-stranded FPs for detecting miR-484.

Strand	Sequence	Number of bases
miR-484	TCAGGCTCAGTCCCCTCCCGAT	22
Quencher55	GTCACATCGGGAGGGGACTGAGCCTGA	27
Signal55	CTCAGTCCCCTCCCGATGTGAC	22
Fuel	GCTCAGTCCCCTCCCGATGTGAC	23
Protector (5'-1; 3'-1)	TCGGGAGGGGACTGA	15
Protector (5'-1; 3'-0)	TCGGGAGGGGACTGAG	16
Protector (5'-1; 3'-2)	TCGGGAGGGGACTG	14
Protector (5'-1; 3'-3)	TCGGGAGGGGACT	13
Protector (5'-0; 3'-0)	ATCGGGAGGGGACTGAG	17
Protector (5'-0; 3'-1)	ATCGGGAGGGGACTGA	16
Protector (5'-0; 3'-2)	ATCGGGAGGGGACTG	15
Protector (5'-0; 3'-3)	ATCGGGAGGGGACT	14
Protector (5'-2; 3'-0)	CGGGAGGGGACTGAG	15
Protector (5'-2; 3'-1)	CGGGAGGGGACTGA	14
Protector (5'-2; 3'-2)	CGGGAGGGGACTG	13
Protector (5'-2; 3'-3)	CGGGAGGGGACT	12

TABLE S4| Dice coefficient for all 5 human liver samples.

No	Dice coefficient	Percentage (%)
Sample 1-2	90.34	5.87
Sample 1-3	85.79	5.23
Sample 2	98.65	43.12
Sample 3	96.43	100
Sample 4	94.21	24.57
Sample 5	98.67	100

MOVIE S1| AFM-based movie of double-stranded SQ in buffer solution.

MOVIE S2| AFM-based movie of double-stranded FP in buffer solution.

MOVIE S3| AFM-based movie of the reaction among double-stranded SQ, FP and miR-484.

MOVIE S4| Dynamic intracellular fluorescence generated by the reaction among double-stranded SQ, FP and miR-484.
